# Supplementary material for: Temperature fluctuations in a changing climate: an ensemble-based experimental approach
Source: Sci Rep. 2017 Mar 21;7:254. doi: 10.1038/s41598-017-00319-0 (PMC5428220; doi:10.1038/s41598-017-00319-0)
Supplement: Supplementary file 1 — Supplementary Information [file 41598_2017_319_MOESM1_ESM.pdf]

Supplementary information for:

Temperature fluctuations in a changing climate: an ensemble-based experimental approach

Vincze, M., Borcia, I. and Harlander, U.

The spectral properties of geostrophic turbulence in the rotating annulus experiment

We analyze the temporal spectral properties of smaller fluctuations as a function of time to determine whether a connection between the scaling properties of the power spectra and ΔT can be established. Power spectra of the temperature fluctuations are calculated in 1000 s-long overlapping windows, with starting points at every 100 s for each ensemble member (following a linear detrending). For each given time section the spectra were averaged over all nine ensemble members. The resulting curves are presented in Fig. S1.a, where the color coding represents the value of ΔT at the beginning of each considered section.

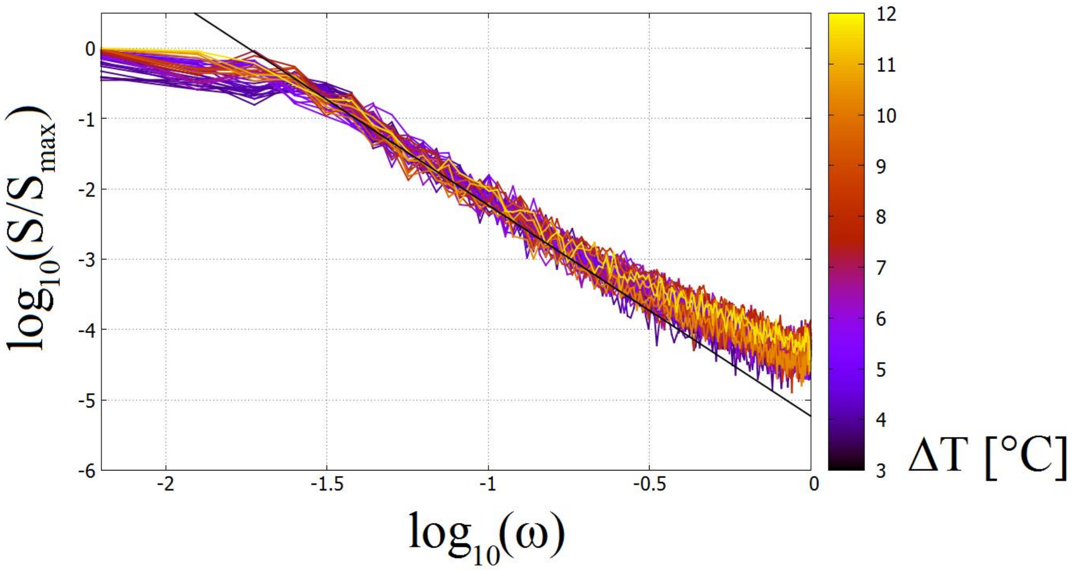

Figure S1. Fourier power spectra in the angular frequency domain of the thermal fluctuation time series $T_i(t)$. The spectra were calculated in 1000 s-long overlapping sections for each ensemble member after a 5th order polynomial detrending, and then the spectra corresponding for the same time interval were ensemble-averaged. The power is rescaled relative to the absolute maximum power, observed throughout the series. The color coding represents the imposed 'meridional' temperature difference values measured at the beginning of each section. The black solid line has a slope of -3 (corresponding to $\beta = 3$ scaling).

In the frequency domain between $\omega = 0.032$ rad/s and 0.32 rad/s (i.e. between -1.5 and 0.5 on the logarithmic scale) the fluctuations appear to follow a power-law $S(\omega) = \omega^{-\beta}$. Linear fits to the curves in this domain yield exponents between $\beta = 3.14$ and 2.57 with an average of 2.77 (standard deviation: 0.13) in good agreement with the theoretical value $\beta = 3$ of isotropic geostrophic (two dimensional) turbulence. This is also in concert with the result that the exponent of the detrended fluctuation analysis (DFA) for mid-timescales is found to be $\delta \approx 2$. (The two exponents are expected to be connected as $\beta = 2\delta - 1$). However, the time- (or, ΔT-) development of the exponents in the different sections does not establish a trend in the slopes of the spectra with changes of the imposed temperature gradient (moreover, the ensemble variance of the spectra at a given time section is also comparable to the temporal variability).

If geostrophic turbulence was 'frozen', one would expect to find the same scaling in the power spectra in the wavenumber domain as in the frequency domain, i.e. $S(k) = k^{-\beta}$. It is therefore of interest to check this hypothesis by plotting the wavenumber spectra, obtained from the infrared images of the single experimental

run, where an infrared camera was used for visualization purposes (see Fig. 2c). From the sequence of images (each separated by 5 minutes in the ‘climate change’ period) the azimuthal thermal fluctuations were extracted from the circular contour at mid-radius (see Fig. S2a and b). Although the spatial resolution is rather limited (the infrared camera captures 640 x 480 pixels, only a fraction of which is actually covered by the water surface), and the high-frequency end of the spectra is covered by white noise, yet, in the domain of intermediate wavenumbers, the slopes indeed appear to follow β = 3 scaling, as visible in Fig. S2c.

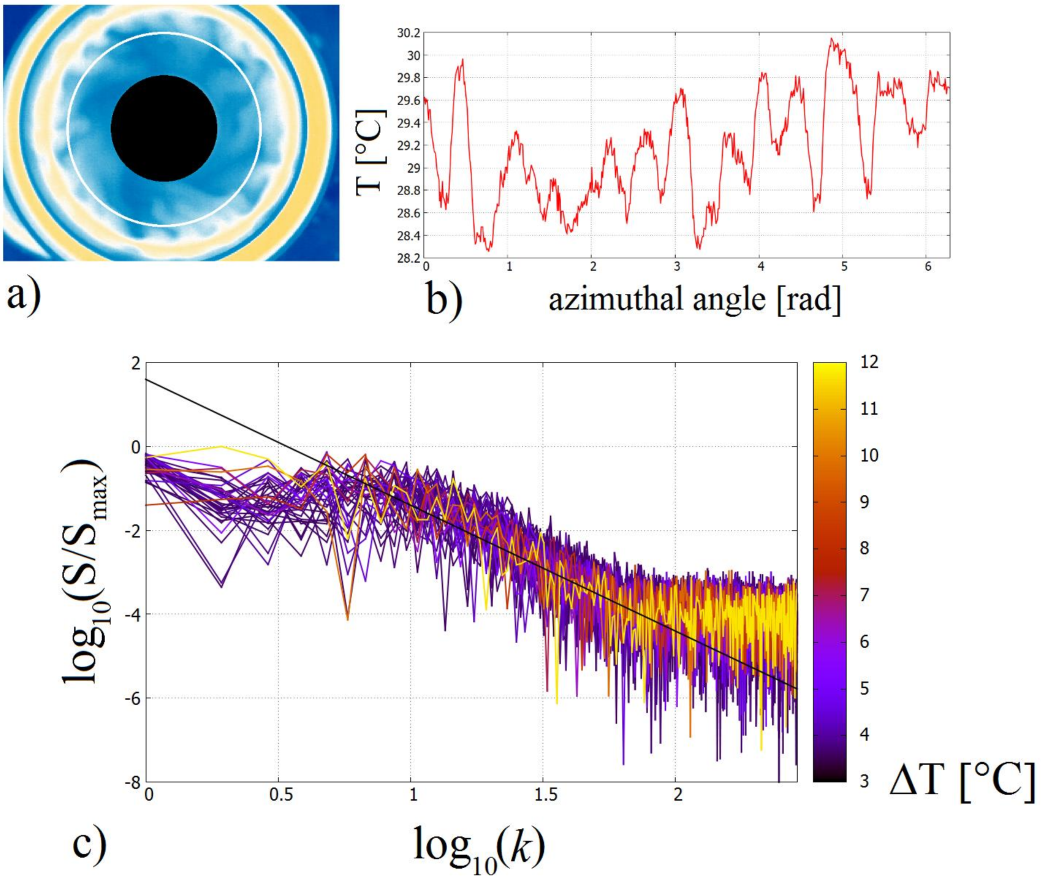

Figure S2. Spatial spectral properties of the thermal fluctuations. (a) A typical infrared thermographic snapshot. The red circle shows the mid-radius contour. (b) The corresponding mid-radius azimuthal temperature profile. (c) Fourier power spectra in the wavenumber (*k*) domain. The color coding represents the imposed temperature difference values corresponding to each snapshot. The black solid line has a slope of -3.

Thus we can conclude, that the geostrophic turbulence in the experiments can be considered ‘frozen’ in the statistical sense. Similarly to the case of frequency spectra, here no significant trend could be established between ΔT and the slopes, implying that the scaling properties of the well-developed geostrophic turbulence did not change significantly throughout the ‘climate change’ period of our experiments.
